# Comprehensive Analysis of 29,464 Cancer Cases and 35,858 Controls to Investigate the Effect of the Cytotoxic T-Lymphocyte Antigen 4 Gene rs231775 A/G Polymorphism on Cancer Risk

**DOI:** 10.3389/fonc.2022.878507

**Published:** 2022-05-04

**Authors:** Hongyuan Wan, Hangsheng Zhou, Yanyan Feng, Yongquan Chen, Lijie Zhu, Yuanyuan Mi

**Affiliations:** ^1^Wuxi Medical College, Jiangnan University, Wuxi, China; ^2^Department of Urology, Affiliated Hospital of Jiangnan University, Wuxi, China; ^3^Wuxi School of Medicine, Jiangnan University, Wuxi, China; ^4^School of Food Science and Technology, Jiangnan University, Wuxi, China

**Keywords:** cancer, cytotoxic T-lymphocyte antigen 4, polymorphism, tumor marker, meta-analysis

## Abstract

In our previous studies, we found that the rs231775 polymorphism of cytotoxic T-lymphocyte antigen 4 (CTLA-4) is associated with risks of different cancer types; however, the association remains controversial and ambiguous, so we conducted an in-depth meta-analysis to verify the association. A complete search of the PubMed, Google Scholar, Embase, Chinese databases, and Web of Science was conducted without regard to language limitations, covering all publications since November 20, 2021. The search criteria for cancer susceptibility associated with the polymorphism in the CTLA-4 gene rs231775 resulted in 87 case-control studies with 29,464 cases and 35,858 controls. The association strength was analyzed using odds ratios and 95% confidence intervals. Overall, we found that the CTLA-4 rs231775 polymorphism may reduce cancer risk. A stratified cancer type analysis showed that CTLA-4 rs231775 polymorphism was a risk factor for colorectal cancer and thyroid cancer; on the other hand, it was a protective factor for breast cancer, liver cancer, cervical cancer, bone cancer, head and neck, and pancreatic cancer. We also classified cancer into five systems and observed an increased association with digestive tract cancer, decreased associations with orthopedic tumors, tumors of the urinary system, and gynecological tumors. In the subgroup based on race, decreased relationships were observed in both Asians and Caucasians. The same decreased association was also shown in the analysis of the source of control analysis. Our present study indicates that the CTLA-4 rs231775 polymorphism contributes to cancer development and aggression.

## Introduction

A major obstacle to increasing life expectancy is cancer, which is the primary cause of death worldwide. Cancer, in 112 of 183 countries, is also estimated to be the first or second leading cause of death before the age of 70 and third or fourth in 23 other countries (1), according to the World Health Organization analyses in 2019 (2). Across the globe, the incidence and mortality of cancer are rising rapidly; this is a result of both increasing longevity and population growth as well as changing patterns in the prevalence and distribution of cancer-causing factors, some of which are associated with social and economic development (1). The development of cancer involves multiple factors, including environmental and genetic factors (3).

One of the most common types of germline variants, the SNPs (single nucleotide polymorphisms), play a key role in human diseases, including cancer (2). Many SNPs associated with human cancer were identified through GWAS (genome-wide association studies) in the past decade (4, 5). Recent studies have noted that the expression levels of nearby genes may be influenced by these cancer risk-associated SNPs (4). Cancer treatment includes traditional surgery, chemotherapy, radiotherapy, and so on. In recent years, immunotherapy has gained more attention (6). The CTLA-4 (cytotoxic T-lymphocyte antigen 4) gene is located on chromosome 2q33 and has four exons (7). Cancer cells can acquire immune regulatory surface proteins like CTLA-4, which suppress the activation of immune cells, such as T cells (3, 8). In the early stages of tumorigenesis, it is possible that CTLA-4 may elevate the threshold of activation of T-cells as it inhibits T cell activation and proliferation. Furthermore, the CTLA-4 competitive binding to B7.1 inhibits IL-2 production and proliferation, both of which are essential in down-regulating T cell activity; in turn, this reduces anti-tumor responses and increases cancer susceptibility (5). Several SNPs in the CTLA-4 gene have been widely reported in tumors and non-tumors, such as rs4553808A/G, rs3087243G/A, rs5742909C/T, rs231726A/a, rs17268364, and rs231775A/G (9–13). The Rs231775 (+49) A/G polymorphism is one of the common SNPs in the CTLA-4 gene (4) and has been extensively reported in many types of cancers. Pavkovic et al. first reported a functional SNP in the CTLA-4 gene (rs231775), indicating that the G-allele frequency was highest among chronic lymphocytic leukemia patients who had developed autoimmune hemolytic anemia (14). Since then, the associations among rs231775 polymorphism and other types of cancer have been reported. In addition, Gouda et al. reported that the genotype (GG) was associated with relatively lower CTLA-4 expression levels than the other genotypes (like GC or CC) (11). To evaluate the effects of the functional SNP and cancer susceptibility, we carried out genotyping analyses among rs231775 A/G in 29,464 cases and 35,858 controls. Here, it would be helpful to explain the role of CTLA-4 in immune response control subsequent to completing its function. This is followed by how the polymorphism affects the function as to whether it increases or decreases the affinity of CTLA-4 to its ligand. The variability in the effect of the polymorphism on susceptibility to cancer warrants more in-depth discussions. Finally, we try to find a few potential explanations, which would add value in this regard.

## Materials and Methods

### Identifying and Evaluating Appropriate Studies

Searches were performed on the Embase, PubMed, Chinese database, Google Scholar, and Web of Science last updated November 20, 2021, using a keyword search that included ‘polymorphism’ or ‘carcinoma’ or ‘CTLA-4’ or ‘cytotoxic T-lymphocyte antigen 4’, or ‘variant’ and ‘cancer’ or ‘tumor’, regardless of language or publication year. These terms led to the retrieval of 592 articles, of which 87 matched the criteria for inclusion. Additionally, we manually searched references of the retrieved or review articles.

### Criteria for Inclusion and Exclusion

The following criteria were required to be included in the review: (a) measured cancer risk in relation to CTLA-4 rs231775 polymorphism; (b) case-control studies; and (c) cases and controls have sufficient genotype numbers. Therefore, we also used the following exclusion criteria: (a) no population was used as control, (b) genotype frequency was not available, and (c) previous publications were duplicated.

### Extraction of Data

Using the selection criteria, the data were extracted independently by two authors. The following data were collected: last name of the first author, publication year, ethnicity, country of origin, cancer type, the total number of cases and controls, source of controls, Hardy-Weinberg equilibrium (HWE) of controls, and genotyping methods.

### Statistical Analysis

The first step was to stratify the subgroups based on cancer type. When a cancer type was reported in only one study, it is classified under the ‘others’ subgroup. In addition, we classified cancer into five systems: digestive tract cancer, orthopedic tumor, tumor of the urinary system, gynecological tumor, and hematological tumor. The ethnicity of the participants was categorized as Asian, Caucasian, and African using two different modes of classification, wherein the source of the control subgroup was analyzed: hospital-based (HB) and population-based (PB). On the basis of genotype frequencies in cases and controls, we calculated OR (odds ratios) with 95% CI (confidence intervals) of the association between CTLA-4 rs231775 polymorphism and the risk for cancer. The overall OR was analyzed using the Z-test (15). Heterogeneity was assessed using chi-square-based Q-tests. The Q-test showed no evidence of heterogeneity among the studies with a P-value greater than 0.05. We used the random-effects model when significant heterogeneity was detected (16); otherwise, the fixed-effects model was applied (16, 17). Using allelic contrast (G-allele vs. A-allele), homozygote comparison (GG vs. AA), dominant genetic model (GG+GA vs. AA), heterozygote comparison (GA vs. AA), and recessive genetic model (GG vs. GA+AA), we investigated the relationship between CTLA-4 rs231775 genetic variants and cancer risk. The Pearson chi-square test was used to calculate HWE in controls at *P*< 0.05. To estimate the likelihood of publication bias, Egger’s regression test and Begg’s funnel plots were used (18). All statistical assessments for this meta-analysis were conducted using Stata software V 11.0 (StataCorp LP, College Station, TX). We calculated the power and sample size of our meta-analysis using PS: Power and Sample Size Calculation (http://www.powerandsamplesize.com/) (19).

### Meta-Regression

The source of publication bias was defined based on a random-effect meta-regression analysis using the publication bias, with publication year as subgroups, ethnicity, source of control, and methods of genotype set as independent variables and the log values regarded as dependent variables (20).

### Bioinformatics Analysis

The expression of CTLA-4 between most types of tumors and para-cancerous tissue is shown from the GEPIA website (http://gepia.cancer-pku.cn/). On the same above-mentioned website, you can also find data about CTLA-4 expression levels in each tumor, which includes overall survival and disease-free survival.

## Results

### Meta-Analysis Study Selection and Characteristics

**Figure 1:**
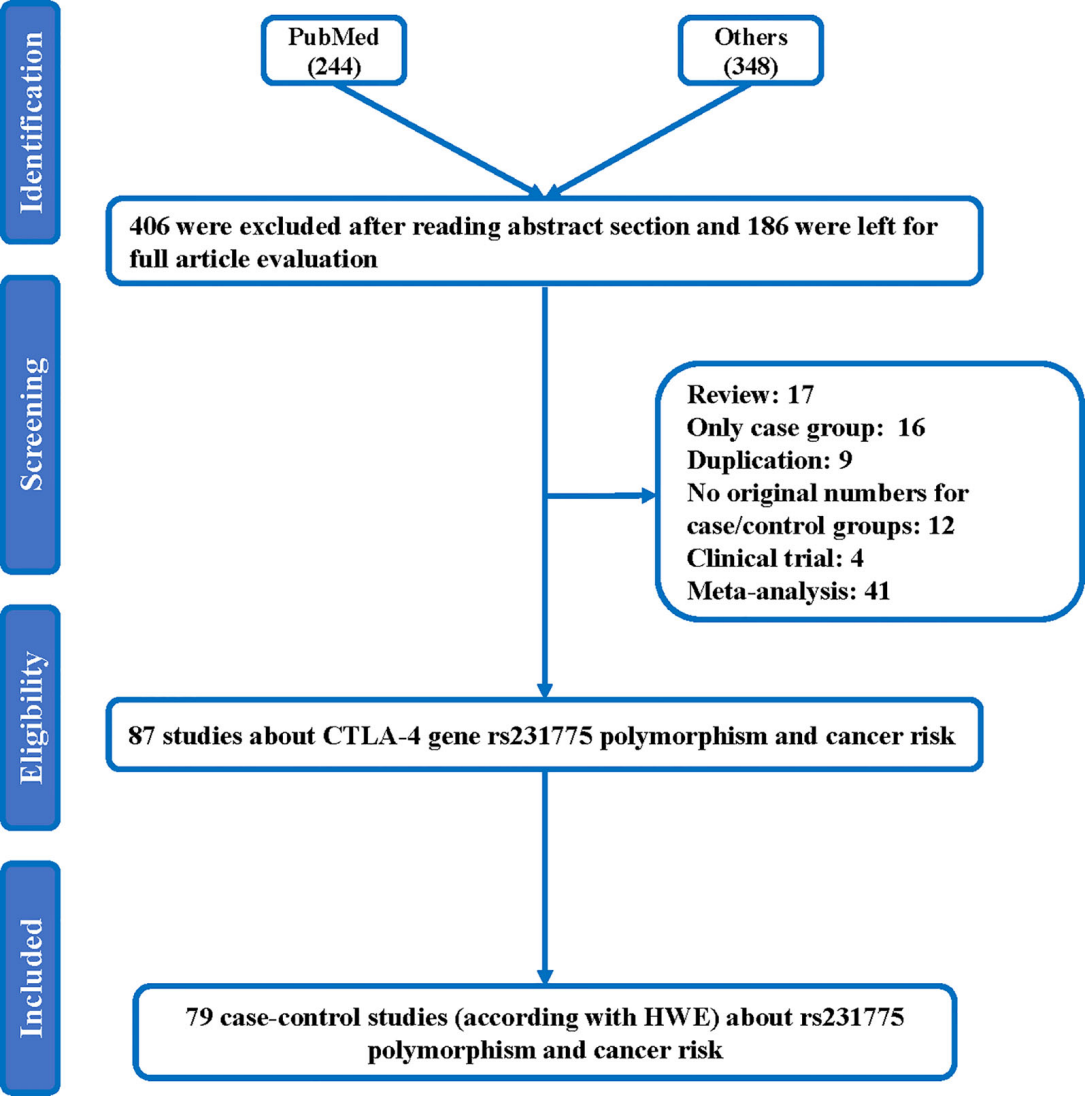
Flowchart illustrating the search strategy used to identify association studies for CTLA-4 rs231775 polymorphism and the total cancer risk.

**Figure 2:**
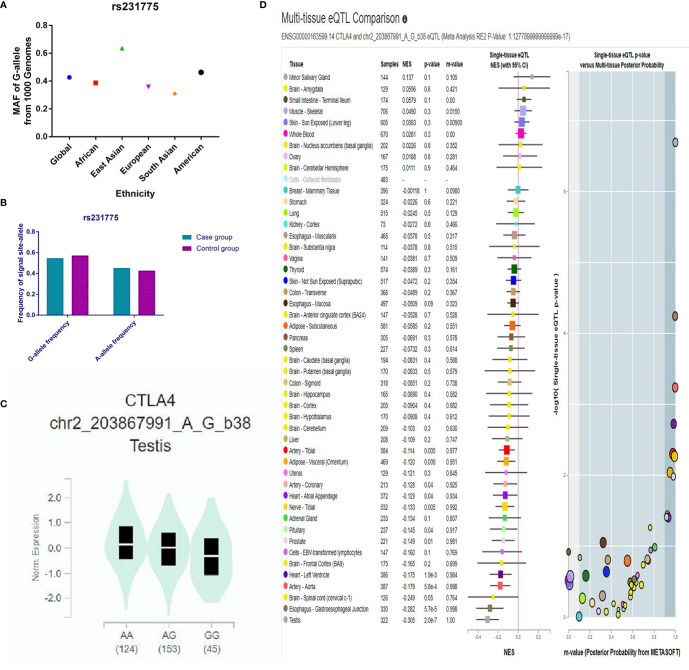
**(A)** The MAF of minor-allele (mutant-allele) for CTLA-4 rs231775 polymorphism from the 1000 Genomes online database. **(B)** The frequency about G-allele or A-allele both in case and control groups. **(C)** The distribution of each genotype from online GTEx Portal (https://www.gtexportal.org/home/). **(D)** The risk frequency of rs231775 polymorphism in several diseases from the TCGA database.

**Figure 3:**
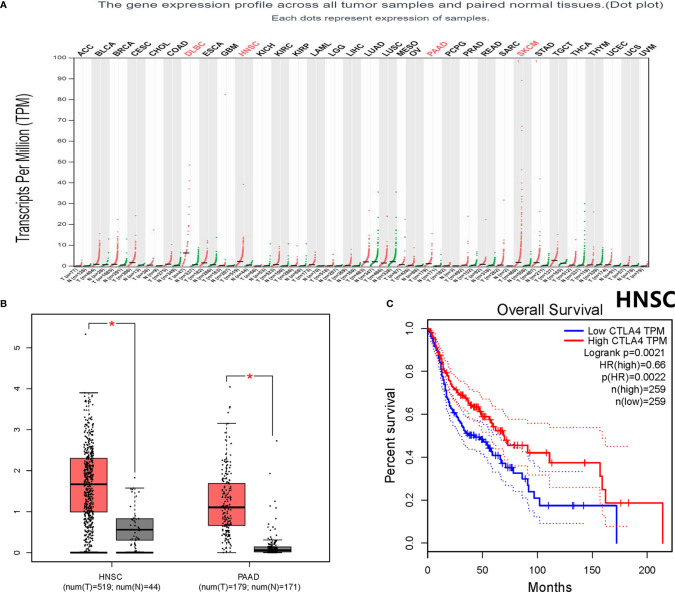
Bioinformatics analysis about the CTLA-4 gene. **(A)** The CTLA-4 gene expression profile across all tumor samples and paired normal tissues. **(B)** CTLA-4 gene expression both in HNSC and PAAD. *P < 0.05. **(C)** Overall survival analysis for HNSC. HR, hazard ratio; ACC, adrenocortical carcinoma; BLCA, bladder urothelial carcinoma; BRCA, breast invasive carcinoma; CESC, cervical squamous cell carcinoma and endocervical adenocarcinoma; CHOL, cholangiocarcinoma; COAD, colon adenocarcinoma; DLBC, lymphoid neoplasm diffuse large B-cell lymphoma; ESCA, esophageal carcinoma; GBM, glioblastoma multiforme; HNSC, head and neck squamous cell carcinoma; KICH, kidney chromophobe; KIRC, kidney renal clear cell carcinoma; KIRP, kidney renal papillary cell carcinoma; LAML, acute myeloid leukemia; LGG, brain lower grade glioma; LIHC, liver hepatocellular carcinoma; LUAD, lung adenocarcinoma; LUSC, lung squamous cell carcinoma; MESO, mesothelioma; OV, ovarian serous cystadenocarcinoma; PAAD, pancreatic adenocarcinoma; PCPG, pheochromocytoma and paraganglioma; PRAD, prostate adenocarcinoma; READ, rectum adenocarcinoma; SARC, sarcoma; SKCM, skin cutaneous melanoma; STAD, stomach adenocarcinoma; TGCT, testicular germ cell tumors; THCA, thyroid carcinoma; THYM, thymoma; UCEC, uterine corpus endometrial carcinoma; UCS, uterine carcinosarcoma; UVM, uveal melanoma.

Throughout different databases, 592 articles were identified, and after a meticulous review, we included 87 varying case-control studies for this study (**Figure 1**). All essential information about included studies is shown in **Table 1**. **Table 1** provides information on the first author, ethnicity, year of publication, cancer type, the numbers of controls and cases, genotyping methods and HWE, and control sources. According to the whole cancer susceptibility search criteria associated with the CTLA-4 rs231775 polymorphism, 87 case-control studies with 35,858 controls and 29,464 cases were retrieved. The controls mainly consisted of healthy populations. Therefore, we have compiled 25 Caucasian, 60 Asian, and 2 African case-control studies for our analyses. The controls in 53 studies came from the source of HB and 34 of PB. We examined the MAF (minor allele frequency) reported for the six major populations globally in the 1000 Genomes Browser (https://www.ncbi.nlm.nih.gov/snp/rs231775) (**Figure 2A**). Moreover, Asians exhibited significantly higher G-allele frequencies than Caucasian individuals both in cases (59.63% vs. 38.19%, P < 0.001) and controls (62.18% vs. 40.36%, P < 0.001) (**Figure 2B**). Third, we used the TCGA (The Cancer Genome Atlas) database to search for trends in the frequency of rs231775 polymorphism; our results indicated that the frequency of AA was relatively high compared to other genotypes, as shown in **Figure 2C**. The polymorphism is associated with prostate, artery, adipose-visceral, heart, nerve, pituitary, testis, and esophagus cancer (https://www.gtexportal.org/home/) (**Figure 2D**). All the controls except for eight studies were genotyped according to HWE. There is significantly more expression of CTLA-4 in tumor tissues than in normal tissue from four kinds of tumors (melanoma of the skin, head and neck squamous cell carcinoma, lymphoid neoplasm diffuse large B-cell lymphoma, pancreatic adenocarcinoma, *P*< 0.05, **Figures 3A, B**). Furthermore, CTLA-4 high expression contributes to a poor overall survival rate in patients with head and neck squamous cell carcinoma (*P*< 0.01) (**Figure 3C**).

**Table 1:** Characteristics of studies of the *CTLA-4* gene rs231775 A/G polymorphism and cancer risk included in our meta-analysis.

First author	Year	Origin	Cancer type (1)	Cancer type (2)	Ethnicity	Source	Case	Control	HWE	Method
Ge et al. (21)	2015	China	Colorectal	Digestive tract cancer	Asian	HB	572	626	0.095	PCR-RFLP
Fan et al. (22)	2012	China	Colorectal	Digestive tract cancer	Asian	HB	291	352	0.059	PCR-RFLP
Qi et al. (23)	2010	China	Colorectal	Digestive tract cancer	Asian	HB	124	407	0.902	PCR-LDR
Hadinia et al. (24)	2007	Iran	Colorectal	Digestive tract cancer	Asian	HB	105	190	0.097	PCR-RFLP
Liu et al. (25)	2015	China	Liver	Digestive tract cancer	Asian	HB	80	78	0.966	PCR-RFLP
Gu et al. (26)	2010	China	Liver	Digestive tract cancer	Asian	HB	367	407	0.902	PCR-LDR
Wang et al. (27)	2015	China	Colorectal	Digestive tract cancer	Asian	HB	311	289	0.001	TaqMan
Dilmec et al. (28)	2008	Turkey	Colorectal	Digestive tract cancer	Caucasian	HB	56	162	0.058	PCR-RFLP
Solerio et al. (29)	2005	Italy	Colorectal	Digestive tract cancer	Caucasian	HB	132	238	0.618	PCR-RFLP
Zou et al. (30)	2019	China	Colorectal	Digestive tract cancer	Asian	PB	979	1299	0.430	SNPscan Kit
Li et al. (31)	2015	China	Colorectal	Digestive tract cancer	Asian	PB	231	325	0.057	PCR-RFLP
Liu et al. (32)	2015	China	Esophageal	Digestive tract cancer	Asian	PB	604	664	0.283	PCR-LDR
Liu et al. (33)	2019	China	Gastric	Digestive tract cancer	Asian	PB	487	1470	0.926	SNPscan Kit
Tang et al. (34)	2015	China	Gastric	Digestive tract cancer	Asian	PB	330	590	0.179	PCR-LDR
Sun et al. (35)	2008	China	Gastric	Digestive tract cancer	Asian	PB	530	530	0.974	PCR-RFLP
Yang et al. (36)	2019	China	Liver	Digestive tract cancer	Asian	PB	575	920	0.893	SNPscan Kit
Hu et al. (37)	2010	China	Liver	Digestive tract cancer	Asian	PB	853	854	0.476	TaqMan
Lang et al. (38)	2012	China	Pancreatic	Digestive tract cancer	Asian	PB	602	651	0.056	PCR-RFLP
Yang et al. (39)	2012	China	Pancreatic	Digestive tract cancer	Asian	PB	368	926	0.828	PCR-RFLP
Cui et al. (40)	2013	China	Colorectal	Digestive tract cancer	Asian	PB	128	205	<0.001	PCR-RFLP
Hou et al. (41)	2010	China	Gastric	Digestive tract cancer	Asian	PB	205	262	0.001	PCR-RFLP
Kucukhuseyin et al. (42)	2015	Turkey	Colorectal	Digestive tract cancer	Caucasian	PB	80	115	0.467	PCR-RFLP
Mahajan et al. (43)	2008	Poland	Gastric	Digestive tract cancer	Caucasian	PB	301	411	0.393	TaqMan
Wagh et al. (44)	2018	Indian	Cervical	Gynecological tumor	Asian	HB	92	57	0.405	PCR-RFLP
Xiong et al. (45)	2014	China	Cervical	Gynecological tumor	Asian	HB	365	421	0.056	TaqMan
Gokhale et al. (46)	2013	Indian	Cervical	Gynecological tumor	Asian	HB	104	162	0.239	PCR-RFLP
Jiang et al. (47)	2011	China	Cervical	Gynecological tumor	Asian	HB	100	110	0.473	PCR-RFLP
Rahimifar et al. (48)	2010	Iran	Cervical	Gynecological tumor	Asian	HB	55	110	0.658	PCR-RFLP
Su et al. (49)	2007	China	Cervical	Gynecological tumor	Asian	HB	139	375	0.351	PCR-RFLP
Pawlak et al. (50)	2010	Poland	Cervical	Gynecological tumor	Caucasian	HB	141	217	0.610	PCR-RFLP
Li et al. (51)	2011	China	Cervical	Gynecological tumor	Asian	PB	314	320	0.339	PCR-RFLP
Hu et al. (37)	2010	China	Cervical	Gynecological tumor	Asian	PB	696	709	0.483	TaqMan
Castro et al. (52)	2009	Sweden	Cervical	Gynecological tumor	Caucasian	PB	953	1715	0.118	Multiplex PCR
Khorshied et al. (53)	2013	Egypt	Lymphoma	Hematological tumors	African	HB	181	200	0.416	PCR-RFLP
Hui et al. (54)	2014	China	Leukemia	Hematological tumors	Asian	HB	86	112	0.137	PCR-RFLP
Cheng et al. (55)	2006	China	Lymphoma	Hematological tumors	Asian	HB	62	250	0.323	PCR-RFLP
Suwalska et al. (56)	2008	Poland	Leukemia	Hematological tumors	Caucasian	HB	170	224	0.524	SNaPshot
Piras et al. (57)	2005	Italy	Lymphoma	Hematological tumors	Caucasian	HB	100	128	0.199	PCR-RFLP
Monne et al. (58)	2004	Italy	Lymphoma	Hematological tumors	Caucasian	HB	44	76	0.837	PCR-RFLP
Pavkovic et al. (59)	2003	Macedonia	Lymphoma	Hematological tumors	Caucasian	HB	130	100	0.533	PCR-RFLP
Liu et al. (60)	2013	China	Lymphoma	Hematological tumors	Asian	PB	291	300	0.163	PCR–LDR
Liu et al. (61)	2011	China	Bone	Orthopedic tumor	Asian	HB	267	282	0.053	PCR-RFLP
Kasamatsu et al. (62)	2020	Japan	Myeloma	Orthopedic tumor	Asian	HB	124	211	0.556	PCR-RFLP
Qin et al. (63)	2017	China	Myeloma	Orthopedic tumor	Asian	HB	86	154	0.201	TaqMan
Aldaiturriaga et al. (64)	2017	Spain	Bone	Orthopedic tumor	Caucasian	HB	66	125	0.101	PCR-RFLP
Feng et al. (65)	2013	China	Bone	Orthopedic tumor	Asian	PB	308	362	0.055	PCR-RFLP
Yang et al. (66)	2012	China	Bone	Orthopedic tumor	Asian	PB	223	302	0.054	PCR-RFLP
Wang et al. (67)	2011	China	Bone	Orthopedic tumor	Asian	PB	205	216	0.130	PCR-RFLP
Karabon et al. (68)	2012	Poland	Bone	Orthopedic tumor	Caucasian	PB	199	368	0.213	PCR-RFLP
Mao et al. (69)	2020	China	Bladder	Tumor of urinary tract	Asian	HB	354	434	0.812	PCR-RFLP
Jaiswal et al. (70)	2014	Indian	Bladder	Tumor of urinary tract	Asian	HB	212	200	0.981	PCR-RFLP
Wang et al. (71)	2013	China	Bladder	Tumor of urinary tract	Asian	HB	300	300	0.005	PCR-RFLP
Lopez et al. (72)	2009	Spain	Renal	Tumor of urinary tract	Caucasian	HB	125	176	0.766	TaqMan
Cozar et al. (73)	2007	Spain	Renal	Tumor of urinary tract	Caucasian	HB	96	176	0.766	PCR-RFLP
Karabon et al. (74)	2017	Poland	Prostate	Tumor of urinary tract	Caucasian	PB	301	301	0.503	PCR-RFLP
Tupikowski et al. (75)	2015	Poland	Renal	Tumor of urinary tract	Caucasian	PB	236	505	0.607	TaqMan
Babteen et al. (76)	2020	Egypt	Breast		African	HB	93	179	0.164	TaqMan
Minhas et al. (77)	2014	Indian	Breast		Asian	HB	250	250	0.197	PCR-RFLP
Wang et al. (78)	2007	China	Breast		Asian	HB	117	148	0.926	PCR-RFLP
Ghaderi et al. (79)	2004	Iran	Breast		Asian	HB	197	151	0.716	PCR-RFLP
Wu et al. (80)	2011	China	Glioma		Asian	HB	653	665	0.841	PCR-LDR
Bharti et al. (81)	2013	Indian	Head and neck		Asian	HB	130	180	0.622	PCR-RFLP
Erfani et al. (82)	2012	Iran	Head and neck		Asian	HB	80	85	0.531	PCR-RFLP
Cheng et al. (83)	2011	China	Head and neck		Asian	HB	205	205	0.054	PCR-RFLP
Xiong et al. 45)	2010	China	Head and neck		Asian	HB	365	421	0.056	PCR-RFLP
Xiao et al. (84)	2009	China	Head and neck		Asian	HB	457	485	0.730	PCR-RFLP
Wong et al. (85)	2006	China	Head and neck		Asian	HB	118	147	0.314	PCR-RFLP
Liu et al. (86)	2015	China	Lung		Asian	HB	231	250	0.059	PCR-RFLP
Khaghanzadeh et al. (87)	2010	Iran	Lung		Asian	HB	123	122	0.763	PCR-RFLP
Abtahi et al. (88)	2018	Iran	Thyroid		Asian	HB	164	100	0.965	PCR-RFLP
Chang et al. (89)	2017	China	Thyroid		Asian	HB	324	350	0.062	PCR-RFLP
Ma et al. (90)	2015	China	Lung		Asian	HB	528	600	0.031	PCR-RFLP
Isitmangil et al. (91)	2016	Turkey	Breast		Caucasian	HB	79	76	0.402	PCR-RFLP
Kammerer et al. (92)	2010	Germany	Head and neck		Caucasian	HB	83	40	0.287	RT-PCR
Queirolo et al. (93)	2013	Italy	Melanoma		Caucasian	HB	14	45	0.802	PCR-RFLP
Antczak et al. (94)	2013	Poland	Lung		Caucasian	HB	71	104	0.001	TaqMan
Chuang et al. (95)	2005	Germany	Thymoma		Caucasian	HB	125	173	0.015	PCR-RFLP
Yu et al. (96)	2015	China	Breast		Asian	PB	376	366	0.962	PCR-RFLP
Li et al. (97)	2012	China	Breast		Asian	PB	576	553	0.739	PCR-RFLP
Sun et al. (35)	2008	China	Breast		Asian	PB	2097	2140	0.053	PCR-RFLP
Sun et al. (35)	2008	China	Head and neck		Asian	PB	1010	1008	0.684	PCR-RFLP
Chen et al. (98)	2017	China	Lung		Asian	PB	520	1028	0.950	SNPscan Kit
Sun et al. (35)	2008	China	Lung		Asian	PB	2205	2153	0.103	PCR-RFLP
Karabon et al. (99)	2011	Poland	Lung		Caucasian	PB	208	324	0.089	PCR-RFLP
Gogas et al. (100)	2010	Greece	Melanoma		Caucasian	PB	286	288	0.465	Multiplex PCR
Bouwhuis et al. (101)	2010	Germany	Melanoma		Caucasian	PB	762	734	0.956	TaqMan
Welsh et al. (102)	2009	USA	Skin		Caucasian	PB	1581	819	0.004	TaqMan

HB, hospital-based; PB, population-based; SOC; source of control; PCR-RFLP, polymerase chain reaction followed by restriction fragment length polymorphism; PCR-LDR, polymerase chain reaction by ligase detection reaction; HWE, Hardy-Weinberg equilibrium of the control group.

### Meta-Analysis

**Figure 4:**
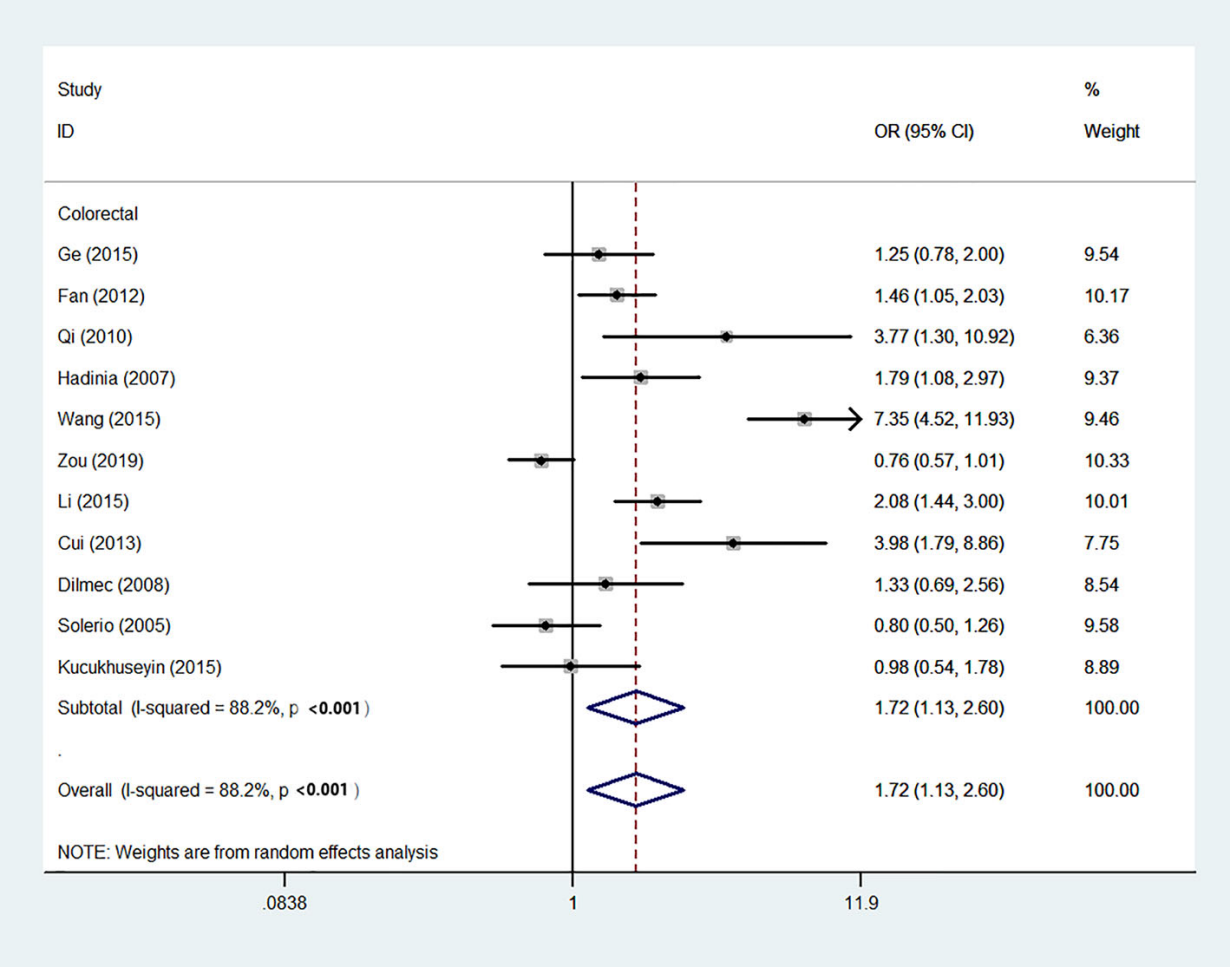
Forest plot of the association between the CTLA-4 gene rs231775 polymorphism and colorectal cancer risk (G-allele vs. A-allele).

**Figure 5:**
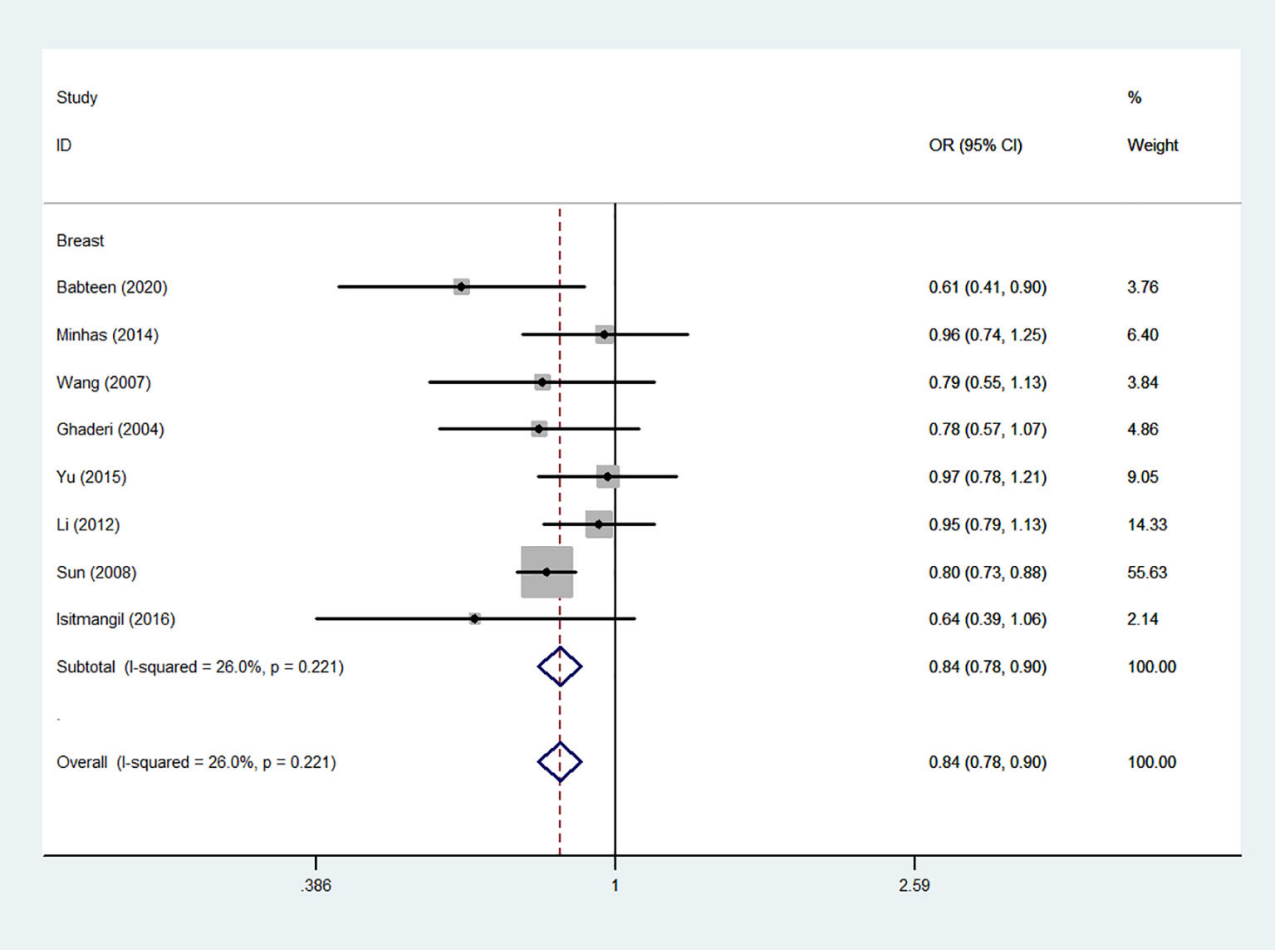
Forest plot of the association between the CTLA-4 gene rs231775 polymorphism and breast cancer risk (G-allele vs. A-allele).

**Figure 6:**
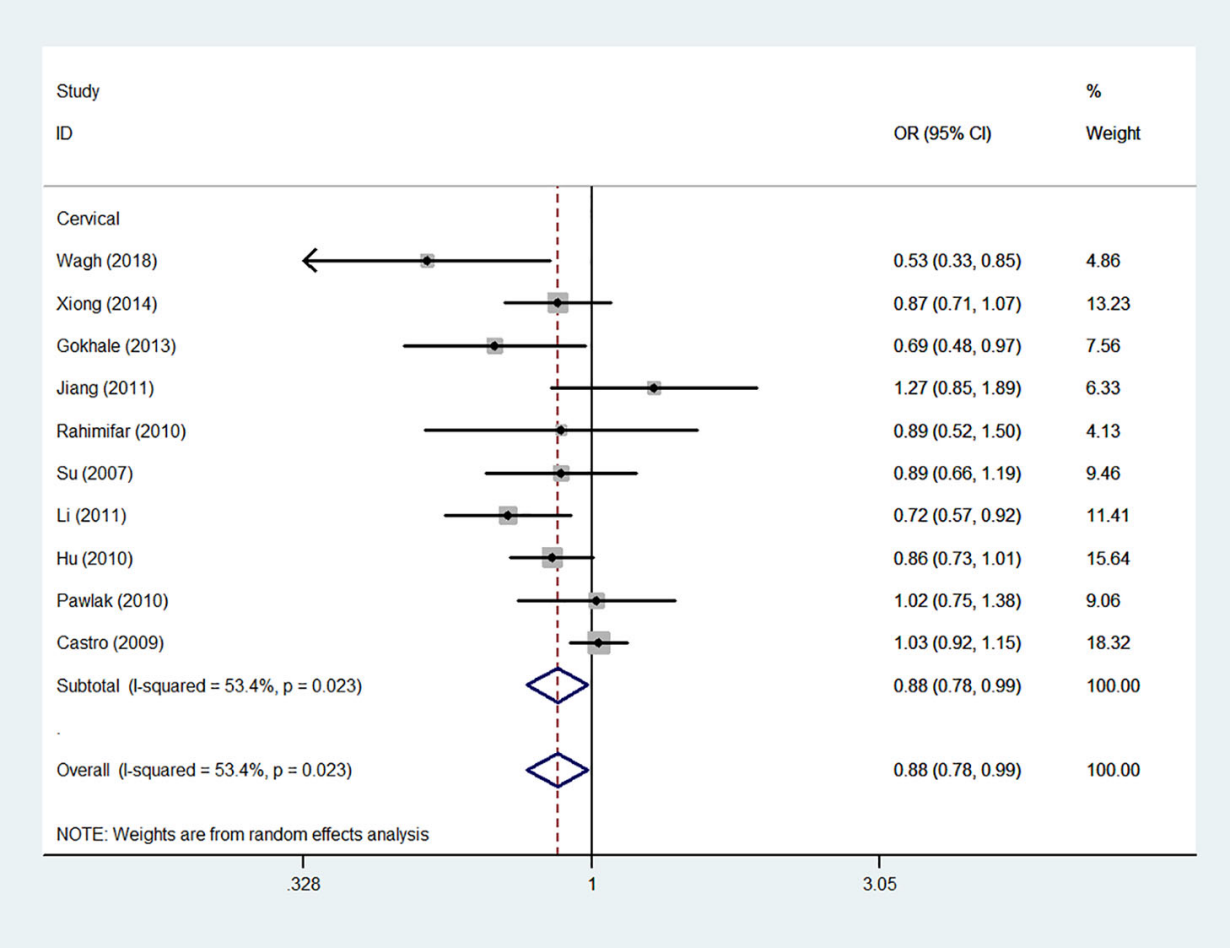
Forest plot of the association between the CTLA-4 gene rs231775 polymorphism and cervical cancer risk (G-allele vs. A-allele).

**Figure 7:**
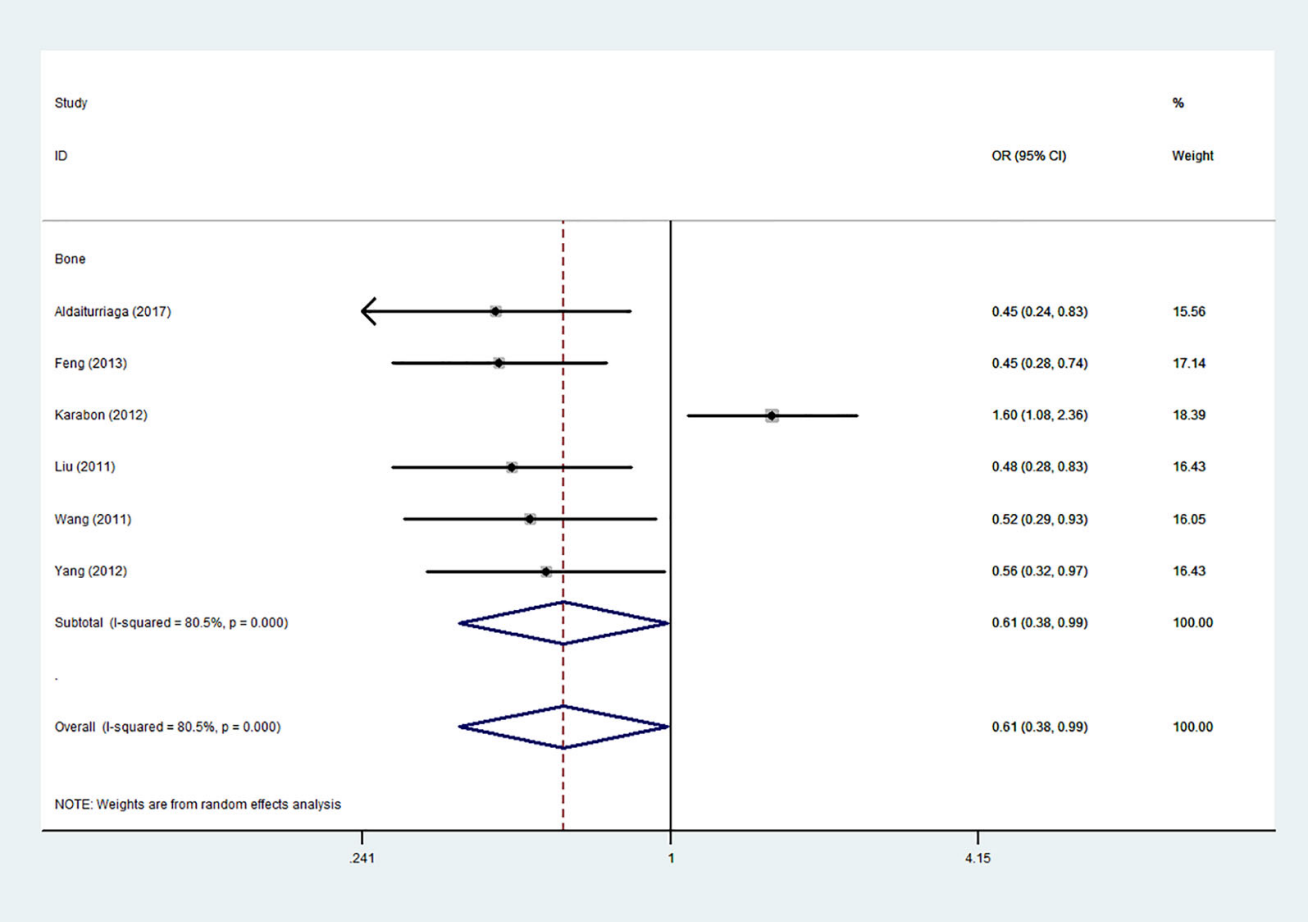
Forest plot of the association between the CTLA-4 gene rs231775 polymorphism and bone cancer risk (G-allele vs. A-allele).

**Figure 8:**
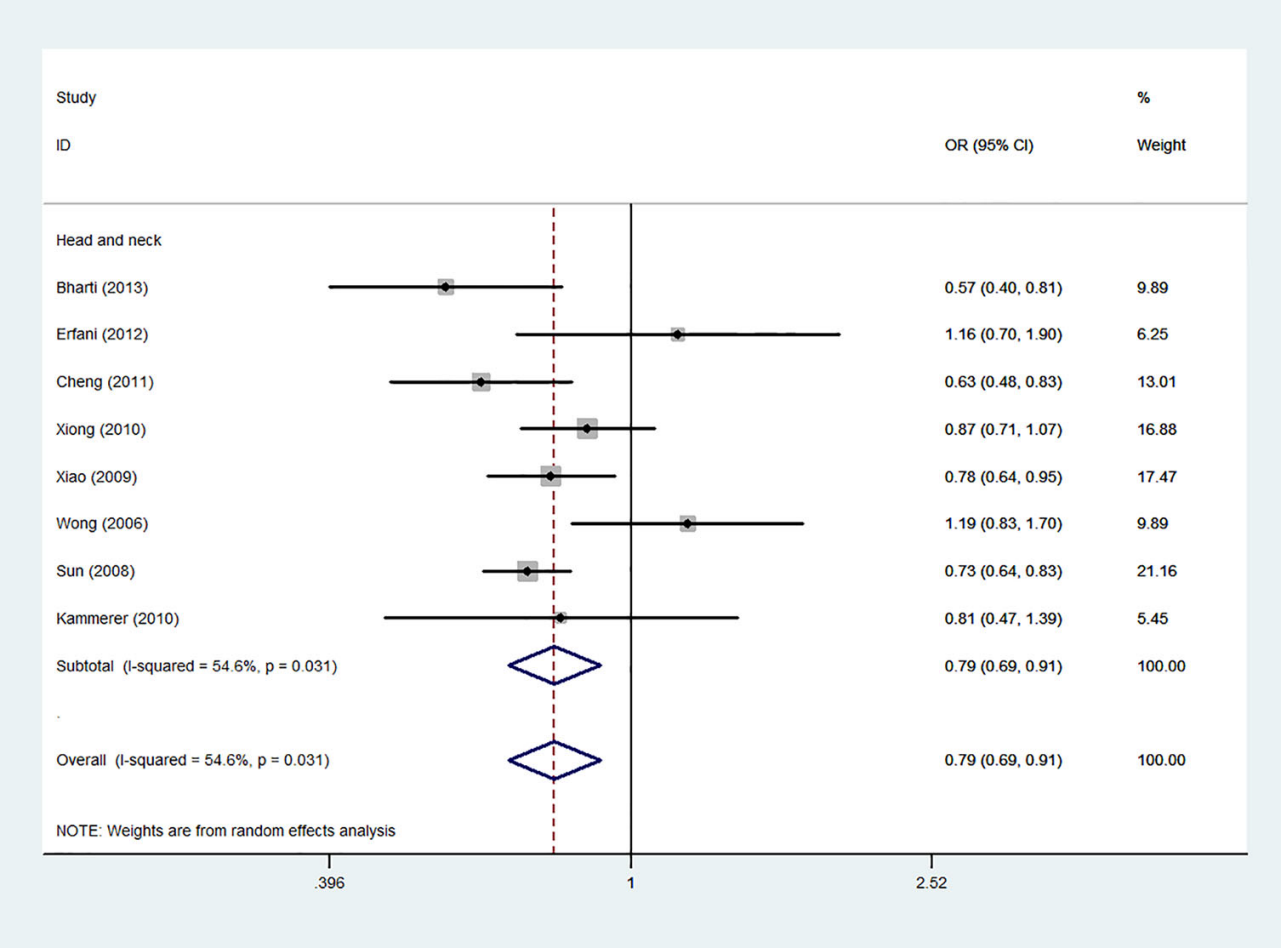
Forest plot of the association between the CTLA-4 gene rs231775 polymorphism and head and neck cancer risk (G-allele vs. A-allele).

Using 29,464 cases and 35,858 controls, the overall risk of CTLA-4 rs231775 is summarized in **Table 2**. CTLA-4 rs231775 polymorphism appears to decrease cancer risk in overall genetic models (G-allele vs. A-allele, OR = 0.94, 95%CI = 0.90-1.00, *P*_heterogeneity_< 0.001, *P* = 0.037; GG vs. AA, OR = 0.86, 95%CI = 0.76-0.96, *P*_heterogeneity_< 0.001, *P* = 0.010; GG vs. GA+AA, OR = 0.88, 95%CI = 0.82-0.94, *P*_heterogeneity_< 0.001, *P* < 0.001). There were significant associations between CTLA-4 polymorphisms and two types of cancers (colorectal cancer: GA vs. AA, OR = 1.72, 95%CI = 1.13-2.60, *P*_heterogeneity_< 0.001, *P* = 0.011; GG+GA vs. AA, OR = 1.52, 95%CI = 1.08-2.15, *P*_heterogeneity_< 0.001, *P* = 0.017, **Figure 4**; thyroid cancer: G-allele vs. A-allele, OR = 1.50, 95%CI = 1.22-1.85, *P*_heterogeneity_= 0.134, *P*< 0.001). On the other hand, significantly decreased associations were detected in six kinds of cancer (breast cancer: G-allele vs. A-allele, OR = 0.84, 95%CI = 0.78-0.90, *P*_heterogeneity_= 0.221, *P*< 0.001, **Figure 5**; liver cancer: G-allele vs. A-allele, OR = 0.89, 95%CI = 0.82-0.98, *P*_heterogeneity_= 0.151, *P* = 0.018; cervical cancer: G-allele vs. A-allele, OR = 0.88, 95%CI = 0.78-0.99, *P*_heterogeneity_= 0.023, *P* = 0.028, **Figure 6**; bone cancer: GG+GA vs. AA, OR = 0.61, 95%CI = 0.38-0.99, *P*_heterogeneity_< 0.001, *P* = 0.044, **Figure 7**; head and neck: G-allele vs. A-allele, OR = 0.79, 95%CI = 0.69-0.91, *P*_heterogeneity_= 0.031, *P* =0.001, **Figure 8**; pancreatic cancer: G-allele vs. A-allele, OR = 0.72, 95%CI = 0.57-0.91, *P*_heterogeneity_= 0.049, *P* = 0.006).

**Table 2:** Stratified analysis of CTLA-4rs231775 A/G variation on cancer susceptibility.

Variables	N	Case/	G-allele vs. A-allele			GA vs. AA			GG vs. AA			GG+GA vs. AA			GG vs. GA+AA			
rs231775 A/G		Control	OR (95%CI)	*P*_h_	*P*	OR (95%CI)	*P*_h_	*P*	OR (95%CI)	*P*_h_	*P*	OR (95%CI)	*P*_h_	*P*	OR (95%CI)	*P*_h_		*P*
Total	87	29464/35858	0.94 (0.90-1.00)	<0.001	0.037	1.01 (0.92-1.12)	<0.001	0.773	0.86 (0.76-0.96)	<0.001	0.010	0.96 (0.87-1.05)	<0.001	0.353	0.88 (0.82-0.94)	<0.001		<0.001
HWE	79	26215/33106	0.93 (0.89-0.98)	≤0.001	0.011	0.97 (0.88-1.06)	≤0.001	0.480	0.83 (0.74-0.93)	≤0.001	0.001	0.92 (0.84-1.01)	≤0.001	0.091	0.88 (0.82-0.94)	≤0.001	≤0.001	
Cancer Type (1)																		
Myeloma	2	210/365	1.17 (0.91-1.51)	0.896	0.209	0.91 (0.53-1.56)	0.138	0.737	1.22 (0.71-2.11)	0.420	0.478	1.05 (0.63-1.75)	0.232	0.858	1.33 (0.94-1.89)	0.578		0.104
Bladder cancer	3	866/934	1.19 (0.73-1.95)	<0.001	0.481	1.24 (1.01-1.51)	0.086	0.040	1.38 (0.41-4.64)	<0.001	0.603	1.24 (0.79-1.97)	0.004	0.353	1.27 (0.42-3.820	0.002		0.668
Breast cancer	8	3785/3863	0.84 (0.78-0.90)	0.221	<0.001	0.86 (0.69-1.07)	0.021	0.169	0.67 (0.57-0.80)	0.134	<0.001	0.81 (0.58-1.37)	0.022	0.042	0.79 (0.71-0.87)	0.370		<0.001
Colorectal cancer	11	3009/4208	1.15 (0.98-1.35)	<0.001	0.094	1.72 (1.13-2.61)	<0.001	0.011	1.24 (0.81-1.90)	<0.001	0.319	1.52 (1.08-2.15)	<0.001	0.017	0.91 (0.71-1.16)	<0.001		0.440
Liver cancer	4	1875/2259	0.89 (0.82-0.98)	0.151	0.018	0.76 (0.62-0.94)	0.870	0.010	0.74 (0.60-0.90)	0.360	0.003	0.75 (0.61-0.91)	0.618	0.004	0.92 (0.81-1.04)	0.164		0.187
Gastric cancer	5	1853/3263	1.07 (0.85-1.35)	<0.001	0.552	1.33 (0.87-2.01)	0.001	0.186	1.15 (0.75-1.80)	0.001	0.513	1.23 (0.81-1.87)	<0.001	0.094	0.94 (0.83-1.06)	0.052		0.325
Cervical cancer	10	2959/4196	0.88 (0.78-0.99)	0.023	0.028	0.88 (0.70-1.10)	0.013	0.257	0.70 (0.52-0.94)	0.006	0.017	0.83 (0.66-1.03)	0.008	0.094	0.83 (0.70-0.99)	0.039		0.043
Thyroid cancer	2	488/450	1.50 (1.22-1.85)	0.134	<0.001	1.96 (1.34-2.87)	0.812	0.001	2.42 (1.48-3.95)	0.400	<0.001	2.13 (1.48-3.07)	0.805	<0.001	1.40 (1.05-1.88)	0.217		0.024
Other cancers	5	3264/2622	0.94 (0.87-1.01)	0.065	0.094	1.00 (0.78-1.29)	0.030	0.991	0.79 (0.7-0.93)	0.109	0.005	0.92 (0.81-1.04)	0.063	0.179	0.88 (0.69-1.11)	0.011		0.279
Lung cancer	7	3886/4581	0.95 (0.73-1.24)	<0.001	0.724	0.98 (0.69-1.40)	<0.001	0.927	0.97 (0.57-1.65)	<0.001	0.901	0.94 (0.62-1.43)	<0.001	0.774	1.01 (0.75-1.35)	<0.001		0.968
Bone cancer	6	1268/1655	0.82 (0.63-1.05)	0.004	0.051	0.63 (0.40-1.00)	0.001	0.051	0.64 (0.38-1.09)	0.001	0.102	0.61 (0.38-0.99)	<0.001	0.044	0.81 (0.69-0.95)	0.125		0.011
Renal cancer	3	457/857	0.85 (0.72-1.00)	0.143	0.056	0.92 (0.71-1.17)	0.125	0.485	0.71 (0.49-1.03)	0.272	0.069	0.85 (0.67-1.08)	0.109	0.185	0.73 (0.52-1.02)	0.485		0.062
Leukemia	2	256/336	0.91 (0.72-1.15)	0.987	0.432	1.10 (0.74-1.66)	0.362	0.634	0.88 (0.54-1.43)	0.592	0.607	1.01 (0.69-1.48)	0.499	0.966	0.78 (0.53-1.14)	0.84		0.197
Head and neck	8	2448/2571	0.79 (0.69-0.91)	0.031	0.001	0.92 (0.68-1.24)	0.004	0.577	0.60 (0.43-0.84)	0.034	0.003	0.80 (0.60-1.06)	0.004	0.123	0.69 (0.53-0.88)	0.017		0.003
Lymphoma	6	808/1054	0.91 (0.63-1.33)	<0.001	0.625	0.99 (0.55-1.77)	<0.001	0.974	1.12 (0.60-2.08)	0.040	0.726	0.96 (0.53-1.76)	<0.001	0.899	1.00 (0.79-1.27)	0.264		0.985
Melanoma	3	1062/1067	1.04 (0.92-1.19)	0.486	0.504	1.14 (0.95-1.37)	0.306	0.165	1.00 (0.76-1.33)	0.767	0.983	1.11 (0.93-1.32)	0.349	0.233	0.95 (0.73-1.23)	0.814		0.706
Pancreatic cancer	2	970/1577	0.72 (0.57-0.91)	0.049	0.006	0.70 (0.53-0.92)	0.766	0.009	0.51 (0.38-0.67)	0.173	<0.001	0.60 (0.46-1.00)	0.347	<0.001	0.67 (0.57-0.79)	0.063		<0.001
Cancer Type (2)																		
Orthopedic tumor	8	1478/2020	0.88 (0.73-1.06)	0.001	0.192	0.68 (0.46-0.99)	0.001	0.048	0.74 (0.47-1.16)	≤0.001	0.192	0.87 (0.62-1.21)	0.006	0.408	0.94 (0.75-1.17)	0.032		0.562
Tumor of urinary tract	7	1624/2002	0.96 (0.76-1.22)	≤0.001	0.755	1.06 (0.86-1.32)	≤0.001	0.553	0.86 (0.53-1.39)	0.002	0.540	0.55 (0.42-0.71)	≤0.001	≤0.001	0.84 (0.56-1.26)	0.009		0.398
Digestive tract cancer	23	8311//11971	1.02 (0.92-1.13)	≤0.001	0.692	1.25 (0.99-1.59)	≤0.001	0.061	0.99 (0.79-1.25)	≤0.001	0.952	1.32 (1.04-1.67)	≤0.001	0.022	0.91 (0.80-1.02)	≤0.001		0.098
Gynecological tumor	10	2959/4196	0.87 (0.78-0.99)	0.023	0.028	0.87 (0.69-1.10)	0.013	0.257	0.70 (0.52-0.94)	0.006	0.017	0.92 (0.74-1.14)	0.014	0.427	0.83 (0.69-0.99)	0.039		0.043
Hematological tumors	8	1064/1390	0.93 (0.71-1.21)≤	0.001	0.577	1.04 (0.68-1.59)	0.001	0.839	1.07 (0.69-1.65)	0.069	0.755	0.82 (0.43-1.57)	≤0.001	0.556	0.93 (0.76-1.14)	0.349		0.480
Ethnicity																		
Asian	60	22851/27839	0.96 (0.90-1.02)	<0.001	0.187	1.06 (0.93-1.20)	<0.001	0.368	0.86 (0.75-1.00)	<0.001	0.053	0.99 (0.88-1.12)	<0.001	0.903	0.87 (0.81-0.95)	<0.001		0.001
African	2	274/379	0.95 (0.41-2.19)	0.001	0.900	0.93 (0.28-3.10)	<0.001	0.910	0.92 (0.25-3.36)	0.027	0.904	0.93 (0.27-3.13)	<0.001	0.902	1.02 (0.60-1.72)	0.208		0.949
Caucasian	25	6339/7640	0.90 (0.81-0.99)	<0.001	0.037	0.95 (0.83-1.09)	<0.001	0.447	0.88 (0.74-1.04)	0.013	0.128	0.90 (0.78-1.04)	<0.001	0.143	0.89 (0.81-0.97)	0.051		0.010
Source of control																		
HB	53	9844/12125	0.94 (0.86-1.03)	<0.001	0.196	1.03 (0.89-1.19)	<0.001	0.684	0.88 (0.73-1.06)	<0.001	0.185	0.97 (0.84-1.12)	<0.001	0.705	0.88 (0.77-1.00)	<0.001		0.046
PB	34	19620/23733	0.93 (0.87-1.00)	<0.001	0.036	0.98 (0.87-1.11)	<0.001	0.761	0.82 (0.71-0.95)	<0.001	0.007	0.93 (0.82-1.05)	<0.001	0.241	0.86 (0.81-0.93)	<0.001		<0.001

P_h_: the value of Q-test for the heterogeneity test; P: Z-test for the statistical significance of the OR.

**Figure 9:**
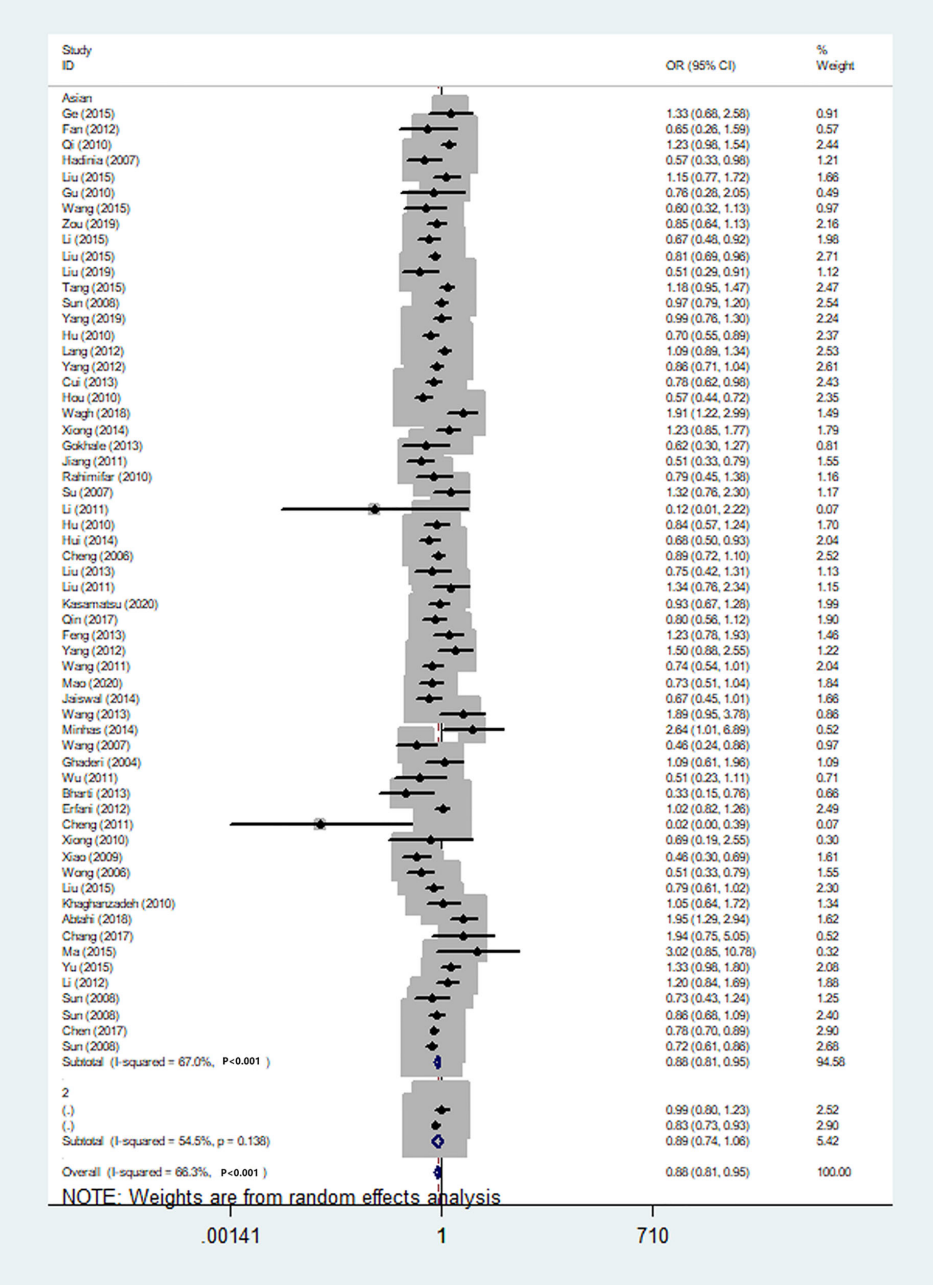
Forest plot of cancer risk associated with the CTLA-4 gene rs231775 polymorphism in Asians (G-allele vs. A-allele model).

**Figure 10:**
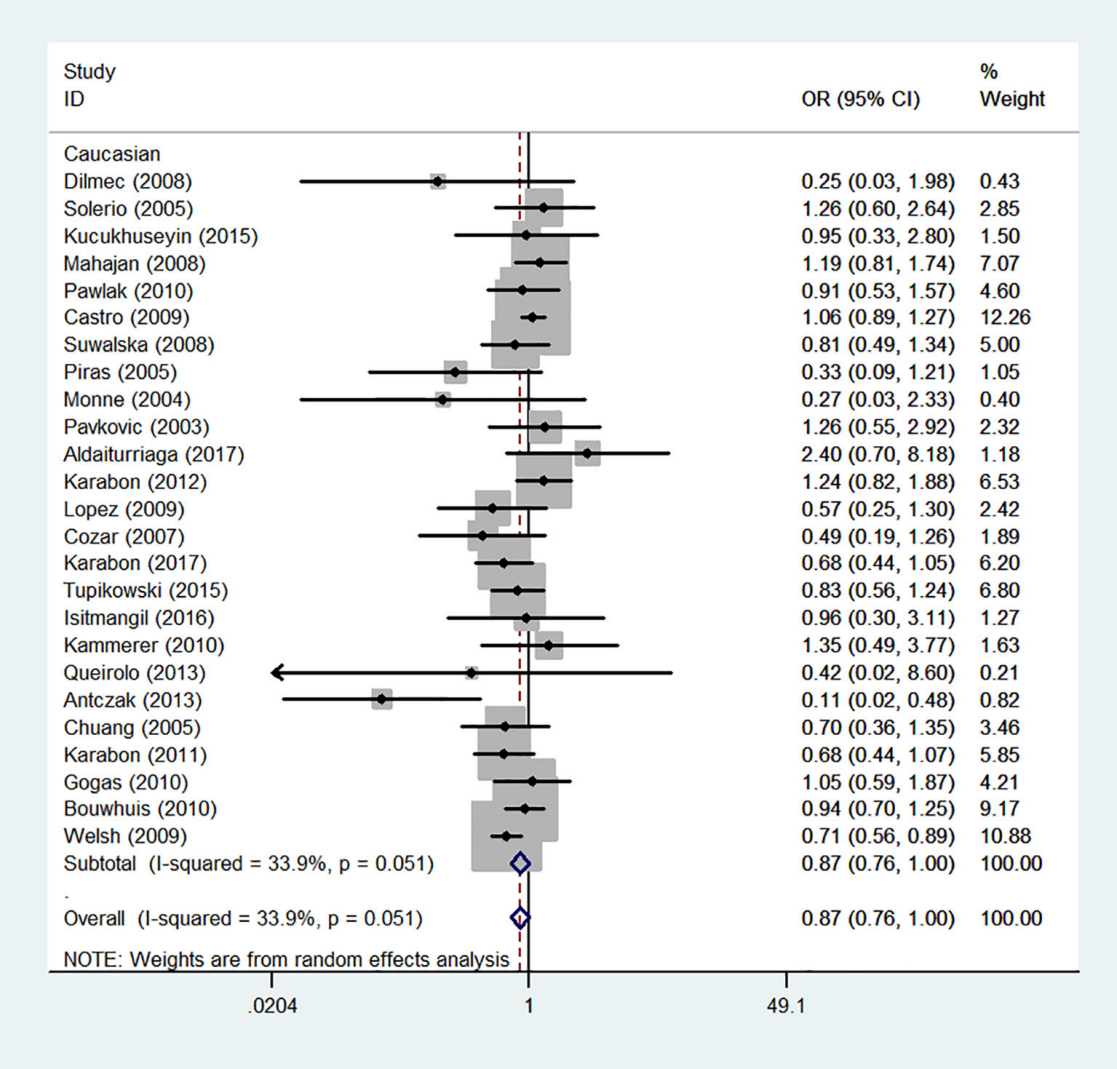
Forest plot of cancer risk associated with the CTLA-4 gene rs231775 polymorphism in Caucasians (G-allele vs. A-allele model).

We also classified tumors into five systems and observed a significant association between the polymorphism and digestive tract cancer (GG+GA vs. AA, OR = 1.32, 95%CI = 1.04-1.67, *P*_heterogeneity_< 0.001, *P* = 0.022), however, decreased associations were observed in three kinds of systems (orthopedic tumor: GG vs. AA: OR = 0.68, 95%CI = 0.46-0.99, *P*_heterogeneity_= 0.001, *P* = 0.048; urinary tract tumor: GG+GA vs. AA, OR = 0.55, 95%CI = 0.42-0.71, *P*_heterogeneity_< 0.001, *P* < 0.001; gynecological tumor: G-allele vs. A-allele, OR = 0.87, 95%CI = 0.78-0.99, *P*_heterogeneity_= 0.023, *P* = 0.028). In spite of variations in the frequency of occurrence of this sequence variant among ethnic groups, decreased cancer risk in both Asian (GG vs. GA+ AA, OR = 0.87, 95%CI = 0.81-0.95, *P*_heterogeneity_< 0.001, *P*=0.001, **Figure 9**) and Caucasian (GG vs. GA+ AA, OR = 0.89, 95%CI = 0.81-0.97, *P*_heterogeneity_= 0.051, *P*=0.010, **Figure 10**) populations was observed. On the basis of stratification by source of control, we evaluated an OR for the rs231775 polymorphism of CTLA-4, and found a decreased association in a recessive genetic model (HB: OR = 0.88, 95%CI = 0.77-1.00, *P*_heterogeneity_< 0.001, *P* = 0.046; PB: OR = 0.86, 95%CI = 0.81-0.93, *P*_heterogeneity_< 0.001, *P <*0.001) (**Table 2**).

### Meta-Regression

**Figure 11:**
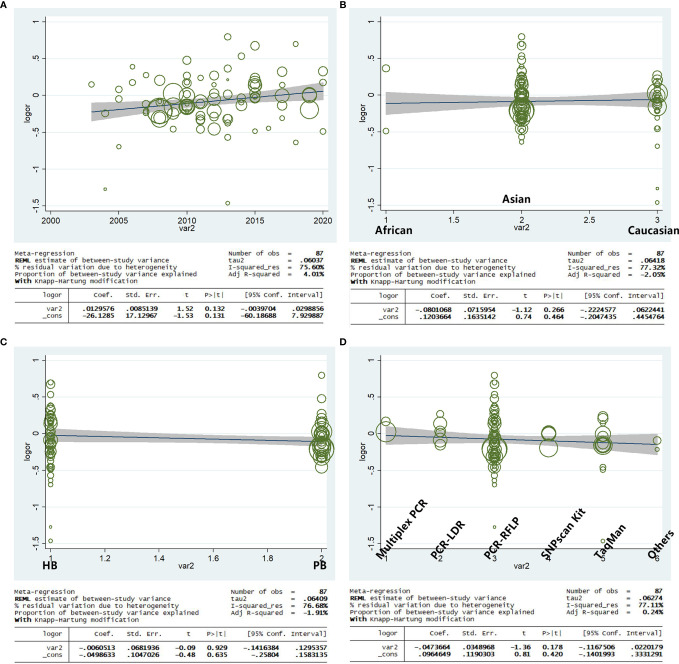
Random-effect meta-regression of log odds ratio vs. publication year **(A)**, regular ethnicity **(B)**, source of control **(C)**, and genotype methods **(D)**, respectively.

Based on the year of publication, ethnicity, genotype methods, and source of control, a meta-regression analysis indicated that there was a significant association for the allele model (A-allele vs. G-allele) with a regression coefficient of 0.131, 0.464, 0.635, and 0.420, respectively, this suggests that the heterogeneity from the rs231775 polymorphism in cancer could not result from the year of publication, ethnicity, source of control, or genotype methods subgroups (**Figures 11A–D**) if the heterogeneity was found in the current study.

## Discussion

Nearly 9 million people die of cancer each year worldwide (103). In the challenge of cancer treatment, immunotherapy has attracted remarkable interest among scientists because of its ability to kill tumor cells directly (14, 104). The Treg cell population expresses a number of immune-modulatory receptors, including CTLA-4, programmed cell death protein 1, and the vascular endothelial growth factor receptor (105). Activated T and Treg cells (106) express CTLA-4. Atkins et al. demonstrated improvement in the rate of survival of non-small cell lung cancer, renal cell carcinoma, melanoma, and head and neck squamous cell cancer by blocking the CTLA-4 immune checkpoint, which showed that the CTLA-4 gene is a promising target gene in the future treatment for cancer (107).

Previously, several meta-analyses were focused on the CTLA-4 polymorphisms, which showed the vital role of CTLA-4 in the susceptibility to many diseases, such as cancer. It was documented that the immune related gene CTLA-4 rs5742909 polymorphism had a significantly increased association with cervical carcinogenesis. Dai et al. found the CTLA-4 rs3087243 polymorphism may reduce breast cancer risk, however, rs4553808 may increase breast cancer risk in different ethnicity or genetic models (108, 109). Another polymorphism rs231775 is the most common SNP that has been reported in many tumors, however, a clear conclusion has not been gained yet despite few meta-analyses (110, 111).

Based on 87 case-control studies, we carried out a meta-analysis, which showed CTLA-4 rs231775 polymorphism plays an important role in cancer risks. According to the results, CTLA-4 rs231775 is strongly associated with the maximum cancer risk. Second, both Asian and Caucasian populations were significantly less likely to develop cancer when individuals carry the rs231775 G-allele. Last, individuals with the rs231775G allele may be at a lower risk for cancer in both HB and PB studies. The results of these studies recommend that the rs231775 polymorphism may contribute to cancer development. Next, based on the stratified cancer type analysis, CTLA-4 rs231775 polymorphism was found to be a risk factor for thyroid cancer and colorectal cancer; that is, in individuals carrying the G-allele, the risk of being diagnosed with cancer is increased; on the other hand, it proved to be a protective factor for liver cancer, breast cancer, cervical cancer, head and neck cancer, bone cancer, and pancreatic cancer, in other words, individuals carrying G-allele may have a lower risk of being diagnosed with cancer. However, no association was detected between this SNP and myeloma, bladder cancer, gastric cancer, lung cancer, renal cancer, leukemia, lymphoma, or melanoma. Some of the reasons why the same gene polymorphism plays different roles in different cancer types may be the difference in the pathogenesis of each kind of cancer, and the same gene and its polymorphism may have different functions and susceptibility.

Gene polymorphisms have the important property of their incidence varying widely across different ethnic populations or races. Based on the subgroup analysis by ethnicity, CTLA-4 rs231775 polymorphism was observed to be significantly associated with lower cancer risks in Asians and Caucasians, but not Africans, suggesting genetic diversity across ethnic groups. This difference can be explained by two factors: genetic and environmental differences among different ethnic groups, and linkage disequilibrium patterns between different populations. Polymorphisms may be related to the presence of closer causal variants in varying populations.

The meta-analysis we performed has certain limitations. To begin with, interactions between gene-environment, gene-gene, or different polymorphic loci of the same gene can modulate the risk for cancer, so researchers should investigate these factors in the future. Moreover, other covariates such as age, sex, family history, environmental factors, cancer stage, and lifestyle should be considered. Furthermore, the control group did not comprise strictly healthy controls. Even so, the meta-analysis we conducted has two advantages. First, data from numerous studies were pooled, significantly increasing the power of the analysis. Second, our selection criteria led to a satisfactory quality of case-control studies that are included in the current meta-analysis. Finally, the strength of the current study as per the software is ‘1’, which indicates the conclusions from our study are convincing and clear.

## Conclusion

The meta-analysis in the current study suggests a significant association between CTLA-4 rs231775 polymorphism and some types of cancer and overall risk for cancer. Consequently, more large-scale studies, which are well-designed, are needed, with a focus on gene-environment and gene-gene interactions. Future research should provide a more comprehensive clarity of the association between the CTLA-4 rs231775 polymorphism and the risk of developing cancer.

## Data Availability Statement

The original contributions presented in the study are included in the article/supplementary material. Further inquiries can be directed to the corresponding authors.

## Ethics Statement

Ethical review and approval was not required for this animal study, in accordance with the local legislation and institutional requirements.

## Author Contributions

HW, YF, and HZ were major contributors in writing the manuscript. HW and YF created all the figures. HZ performed the literature search. LZ, YC and YM made substantial contributions to the design of the manuscript and revised it critically for important intellectual content. All authors have read and approved the final version of this manuscript.

## Funding

This work was supported by National Natural Science Foundation (No. 81802576), Wuxi Commission of Health and Family Planning (No. T202024, J202012, Z202011), the Science and Technology Development Fund of Wuxi (No. WX18IIAN024, N20202021), and Jiangnan University Wuxi School of Medicine (No. 1286010242190070) and Wuxi “Taihu Talent Program”-High-end Talent in Medical and Healthentalent plan of Taihu Lake in Wuxi (Double Hundred Medical Youth Professionals Program) from Health Committee of Wuxi (No. BJ2020061).

## Conflict of Interest

The authors declare that the research was conducted in the absence of any commercial or financial relationships that could be construed as a potential conflict of interest.

## Publisher’s Note

All claims expressed in this article are solely those of the authors and do not necessarily represent those of their affiliated organizations, or those of the publisher, the editors and the reviewers. Any product that may be evaluated in this article, or claim that may be made by its manufacturer, is not guaranteed or endorsed by the publisher.
